# The intelligence quotient of school aged children delivered by cesarean section and vaginal delivery

**Published:** 2010

**Authors:** Nayereh Khadem, Talaat Khadivzadeh

**Affiliations:** * Associated Professor and Head of Women’s Health Research Center, Mashhad University of Medical Sciences, Mashhad, Iran; ** Student and Member of Women Health Research Center, Mashhad University of Medical Sciences, Mashhad, Iran

**Keywords:** Intelligence quotient, school aged children, cesarean and vaginal delivery

## Abstract

**BACKGROUND:**

There has always been an asking question with physicians and health staff whether delivery mode can effect on child intelligence. This study was conducted to compare the intelligence quotient (IQ) of school aged children delivered by cesarean section and vaginal delivery in Mashhad, Iran.

**METHODS:**

This study conducted in two stages; a cross-sectional section in which 5000 randomly selected children, who were 6-7 years old, attended at 10 Cognitive Examination Posts in Mashhad. The examination was performed by the Exceptional Education and Training Institute affiliated to Ministry of Education for all 6-7 years old children at the entry to the primary school. At the second stage, we selected two matched groups of 189 children who delivered by cesarean section or spontaneous vaginal delivery and then compared their IQ scores.

**RESULTS:**

The cesarean delivery group had significantly higher IQ test scores. Maternal and paternal educational levels were related to children’s IQ scores. After adjusting of maternal and paternal education, maternal age and parity, there was not any significant difference between IQ scores of cesarean delivery and natural vaginal delivery groups 101(3.67) vs. 100.7(4.28).

**CONCLUSIONS:**

Based on our findings, the association between cesarean deliveries with better cognitive development in children cannot be supported.

In recent years, cesarean rate was increasing in the world.^1^–^3^ One of the most important reasons was the elective cesarean.^4^,^5^ In the last Department of Human Services (DHS) survey in Iran, the caesarean birth rate was almost 40%.^6^ In a study in Isfahan, this rate reported as 52.8%.^7^ Several studies showed a great number of mothers and even some of physicians and midwives who had a positive attitude toward the cesarean section.^8^–^12^

Despite the lack of evidences in relationship between delivery mode and children’s intelligence quotient (IQ), this belief was common between many people and also many physicians and health staff that brain and cognitive function of children are negatively affected by pressures during passing the birth canal and birth traumas. Previous studies in our country showed that many mothers believe that vaginal deliveries may had negative influences on intellectual performance of their neonates.^13^,^14^ In the study of Seyed Noori et al, 35.2% of mothers believed that children born by cesarean delivery were more intelligent.^14^ The previous studies did not show such results. However, further cognitive outcomes in follow-up studies of infants delivered by cesarean section or vaginally are still ambiguous. In Hohlweg-Majert et al study, the intelligence quotient of children delivered by cesarean obtained 5.7 point scores more than children delivered vaginally which was not statistically significant.^15^

Seidman et al, after adjustment of confounding factors, found that the intelligence quotient of cesarean born children was lower than spontaneous delivery group.^16^ In Eid et al study on intellectual performance of 8738 males at the age of 18, in an unadjusted analysis, cephalic presented males gained higher intellectual scores if their mothers had cesarean delivery instead of vaginal delivery, on the other hand, in adjusted analysis, the scores were slightly lower among cephalic presented males who were delivered by cesarean in comparison with vaginal delivery that this might be due to more pathologic conditions during their mothers’ pregnancy and child birth.^17^

The outcomes of delivery modes on infants and child’s cognitive function were not investigated through prospective studies. In this regard, few existed studies showed opposite results. In those studies, the effects of confounding factors that could be in relation with both mode of the delivery and children’s intelligence quotient were not investigated.

As the presence of a doubt regarding to the association of delivery mode with long term cognitive development and an increasing high intention in cesarean deliveries in our country, this study was conducted to determine the intelligence quotient of school aged children and its comparison between children delivered by cesarean section and vaginal delivery in Mashhad, Iran.

## Methods

This study conducted in two stages. At the first cross-sectional stage, 5000 children with age of 6-7, who attended at 10 Cognitive Examination Posts in Mashhad, were randomly selected. Assessment of intelligence quotient was performed by the Exceptional Education and Training Institute affiliated to Ministry of Education for all 6-7 years old children at the entry to the primary school.

The children with history of meningitis, fever and compulsion, brain trauma, disabled children, children born after complicated pregnancies and also other non-natives of Iran were excluded from the study. The data about individual characteristics, pregnancy, and delivery histories were gathered through interviewing with mothers. The children’s cognitive examination included the raven colored progressive matrices which estimated the children’s ability for visual observation and interpretation.

Ravan test was based on some tables and pictures that had a logical order. It’s validity in assessment of general intelligence quotient was very high.^18^

The Wechsler Intelligence Scale Test (WISC) and Leither International Performance Scale were performed for children with intelligence quotient of less than 12. The Wechsler Revised Scale which included 11 subscales was normalized by Shahim in order to use for Iranian children.^19^ The International Practical Leither test was a non verbal test administered using cubic tools. This test was applied for identifying the children with verbal and speaking disorder or problem in verbal communications.^20^

The IQ was calculated dividing the gained scores to chronological age and multiple it in 100.^21^ The mean scores of standardized Raven test were identified and compared based on the delivery modes.

At the second case-control stage of the study, in order to assess the relationship between the mode of delivery and IQ, two matched groups of 189 children born by cesarean section and spontaneous vaginal delivery were selected. Two groups of children were similar in terms of maternal age, parity, paternal education, birth rank, and use of kindergarten and child’s rank. All of the children in two groups had birth weight of 2500 grams or more and delivered through vertex presentation. Children with history of labor course of more than 12 hours or problems at the birth and who lived with one or none of their parents were excluded at this stage.

Data were analyzed using student t-test and one-way analysis of variance to compare the means of intelligence scores of different groups of children and chi-square test for analyzing the relationship between qualitative data.

The ethical committee of Mashhad University of Medical Sciences approved the study.

## Results

The age of mothers in 6% of cases were 15-20, in 42.3% 21-30, in 46.9% 31-40 and in 10.20% 41-50 years old. Table 1 shows the distribution level of mothers and fathers education. Birth rank of 29% of children was first, 21.7% was second, 19.8% was third, 27.5% was forth and 2% had undefined birth rank. 6.1% of children lived only with one of the parents (either mother or father). At the birth time, the presentation of 2.6% of them was breech and 87.6% cephalic. In 9.8% of children, the presentation was not defined. Among 5000 children, the delivery mode was cesarean section in 10.4% and vaginal delivery in 83.48%. The frequency of instrumental delivery was 3.92%.

**Table 1:** Mean of children’s intelligent quotient scores based on maternal and paternal education

Parents’ educational level	Mother			Father		
	No.	Percent	Mean (SD) Children’s IQ	No.	Percent	Mean (SD) Children’s IQ
Uneducated	354	6.61	5.03 (97.95)	532	6.28	6.81 (96.15)
Primary school	1606	6.45	6.47 (99.11)	231	8.82	5.32 (98.91)
Guidance school	1391	12.21	5.23 (100.13)	1401	12.6	5.48 (102.51)
High school	1075	15.12	4.36 (102.26)	752	15.18	37.4 (103.37)
Associate degree	238	14.58	3.98 (107.19)	98	14.7	4.17 (105.21)
Bachelor of science and more	232	15.52	5.18 (105.24)	71	18.18	6.62 (108.55)

p = 0.000

**Figure 1:**
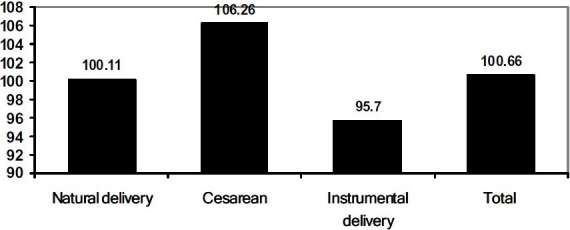
Intelligent quotient of 5000 children of 6-7 years old based on the delivery mode

In the primary analysis, the mean (SD) of children’s intelligence quotient was 100.66(4.27). The findings showed a significant difference between the IQ scores of children born through cesarean and natural vaginal delivery (p = 0.001), cesarean and instrumental delivery (p = 0.001), and natural vaginal delivery and instrumental delivery (p = 0.001). The children born through cesarean had the highest and the children born through instrumental delivery had the lowest IQ scores (Figure 1).

In order to identify the effects of confounding factors on IQ, the effect of other factors were investigated. Based on M-ANOVA, the maternal and paternal education had a high relationship with children’s IQ (p = 0.001). Using Tukey test, the children’s IQ scores were significantly different between all the maternal educational levels, and also between all the paternal educational levels Table 1. There was also a significant relationship between maternal age and children’s IQ. The mean score of children’s IQ based on the maternal age in groups of 15-20, 20-30, 30-40 and more than 40 years was equal to 98.67, 101.85, 101.75 and 97.2, respectively. There was a significant difference between the IQ scores of children with maternal age of 20-40 years compared to less than 20 (p = 0.002) or more than 40 (p = 0.003) years old.

There was a significant relationship between children’s IQ scores and their birth ranks. The IQ scores of children based on the rank were as following: the first rank 102.21, the second rank 101.95, the third rank 101.61 and the fourth rank and more 100.14. Using Tukey test, the IQ scores of children of first rank were significantly higher than the third (p = 0.001) and the forth and more (p = 0.000). In addition, there was a significant relationship between the IQ scores of children of the second rank with children of the third (p = 0.001) and the fourth and more (p = 0.001). The mean of intelligence quotient score of children who lived with one or none of their parents was equal to 93.5 6.8) and was significantly lower than the children who lived with both of their parents.

In order to control the confounding factors, at the second stage of our study, from 5000 participants of the first stage, 189 children from cesarean section group and 189 matched children from natural vaginal delivery group were selected. Data were controlled for maternal and paternal education, maternal age and parity and child rank. After adjusting of confounding factors, there was not any significant difference between IQ scores of cesarean delivery and spontaneous vaginal delivery groups (Table 2).

**Table 2:** The comparison of intelligent quotient scores of children born through natural vaginal delivery and cesarean section after adjusting of confounding factors

Delivery mode	No.	Percent	Mean (SD) Children’s IQ
Natural delivery	189	50	4.28 (100.7)
Cesarean	189	50	3.67 (101)
Total	372	100	4.33 (100.4)

T = 0.73; p = 0.46

## Discussion

In this study, the intelligence quotient of children of 6-7 years age who want to enter to elementary schools was assessed and the difference between the two groups of children born through natural vaginal delivery and cesarean section was compared.

At the primary analysis, the intelligence quotient score of children in cesarean delivery group was significantly higher than vaginal delivery group. Our findings also showed that the IQ score of children was in a high positive relationship with maternal and paternal education. The rate of cesarean section was also higher in more educated mothers and fathers. The rate of the cesarean in mothers with academic education was 2.5 times more than this rate in uneducated mothers. As a result, the higher IQ in children might be in association with more intelligence of their educated mothers and not in association with the mode of delivery. However, this difference between the intelligence quotient of cesarean and natural delivery group was not observed after adjusting of confounding factors such as maternal and paternal education, maternal age, and child’s birth rank. These findings were in accordance with some other controlled studies which evaluated the relationship between delivery modes and intelligence quotient. In a collaborative study by Wesley et al there was no significant difference in standardized intelligence scores according to the mode of delivery in 5 year-old children.^22^

Cesarean section had no pay off even for the children born with breech presentation and for preterm neonates in previous studies. In the study of Ulander et al need for specific additional teaching at school among infants delivered vaginally or by cesarean in breech or cephalic presentation was similar.^23^ Two other studies failed to present any effect of delivery method among breech neonates on their intellectual performance.^24^,^25^ Litt et al study on neural development of very low birth weight infants at the age of 2, showed no clinically benefits for them delivered by cesarean in comparison to whom delivered vaginally.^26^

In the study of Seidman et al on people who were examined at the age of 17, the mean of IQ score of cesarean section and vaginal delivery groups were 105.4(0.4) and 105.4(0.1), respectively that had no significant differences and were lower than instrumental delivery groups. In their study, after adjustment for confounding factors, the IQ score of the cesarean section group was 103.7(0.1) which was significantly lower than that of spontaneous delivery group with the score of 105.7(0.1).^16^

The study of Romer et al on school aged children showed that those who born vaginally after a 12 hours or more course of labor had lower intelligence quotient than those born vaginally after a shorter course of labor. In comparison with children who had been delivered by cesarean section, who were the more appropriate control group, they had significantly lower intelligence scores which indicated the prolonged and obstructed labor which might adversely affect IQ. In this condition, the cesarean section is preferred and can maintain the children from morbidities and mental sequel and can be performed with safety for mothers and fetuses.^27^ In the study of Hohlweg-Majert et al, no significant relationship was found between the type of delivery and intelligence quotient. Base on Kramer-Binet Intelligence Test, a mean IQ of 114.4 was found for those delivered by section, 116.6 for those by forceps, and 117.7 for those delivered by vacuum. The spontaneous-born children had IQ of 108.7.^15^

As the present study was retrospective, we had no access to precise data about pregnancy and delivery, but in controlling the confounding factors in analysis of two groups a, we excluded all the people who were in doubt for any complication based on mothers’ declaration. Consequently, we included only the group of mothers who their cesarean delivery was elective. Although initially analysis showed that cesarean section was associated with higher IQ in the babies of mothers without complicated pregnancies, this effect was not observed after controlling the confounding factors. Wellcontrolled studies did not show any increase in intelligence of children delivered by cesarean section. Based on our findings, the association between cesarean deliveries with better cognitive development in children cannot be supported.

It should be considered that our study was done on the children whose mothers had no complicated pregnancy, labor and their conditions were very similar to the mothers who have chosen elective cesarean without medical indication. These findings cannot be generalized to the mothers who underwent cesarean section or vaginal delivery due to the complicated pregnancies or deliveries or medical indications. Findings of the present study can be applied into antenatal counseling and education programs about the mode of delivery. Maternity care providers should identify and correct the mothers and their relatives’ beliefs and attitudes on the vaginal delivery and cesarean section. Mothers should be empowered in making an informed decision which provides the safety of mothers and children in a more ideal situation.

The authors declare no conflict of interest in this study.
